# Evidence for a developing plate boundary in the western Mediterranean

**DOI:** 10.1038/s41467-022-31895-z

**Published:** 2022-08-15

**Authors:** Laura Gómez de la Peña, César R. Ranero, Eulàlia Gràcia, Guillermo Booth-Rea, José Miguel Azañón, Umberta Tinivella, Abdelkarim Yelles-Chaouche

**Affiliations:** 1 Barcelona Center for Subsurface Imaging, Instituto de Ciencias del Mar, CSIC, Barcelona, Spain; 2 ICREA, Barcelona, Spain; 3 Facultad de Ciencias, Universidad de Granada, Granada, Spain; 4 Instituto Andaluz de Ciencias de la Tierra, CSIC, Granada, Spain; 5 Istituto Nazionale di Ocenagrafia e di Geofisica Sperimentale, Trieste, Italy; 6 Centre de Recherche en Astronomie, Astrophysique et Géophysique, Algiers, Algeria

**Keywords:** Natural hazards, Geodynamics, Tectonics

## Abstract

The current diffuse-strain model of the collision between Africa and Eurasia in the western Mediterranean predicts a broad region with deformation distributed among numerous faults and moderate-magnitude seismicity. However, the model is untested because most deformation occurs underwater, at poorly characterized faults of undetermined slip. Here we assess the diffuse-strain model analysing two active offshore fault systems associated with the most prominent seafloor relief in the region. We use pre-stack depth migrated seismic images to estimate, for the first time, the total Plio-Holocene slip of the right-lateral Yusuf and reverse Alboran Ridge structurally linked fault system. We show that kinematic restoration of deformational structures predicts a slip of 16 ± 4.7 km for the Alboran Ridge Fault and a minimum of 12 km for the Yusuf Fault. Thus, this fault system forms a well-defined narrow plate boundary that has absorbed most of the 24 ± 5 km Plio-Holocene Africa-Eurasia convergence and represents an underappreciated hazard.

## Abstract (web summary)

Conventional models propose multiple fault systems across a diffuse deformation zone absorbing plate convergence in the western Mediterranean. Here the authors show new data supporting the active development of a single plate boundary fault system, representing an underappreciated seismic and tsunami hazard.

## Introduction

**Fig. 1:**
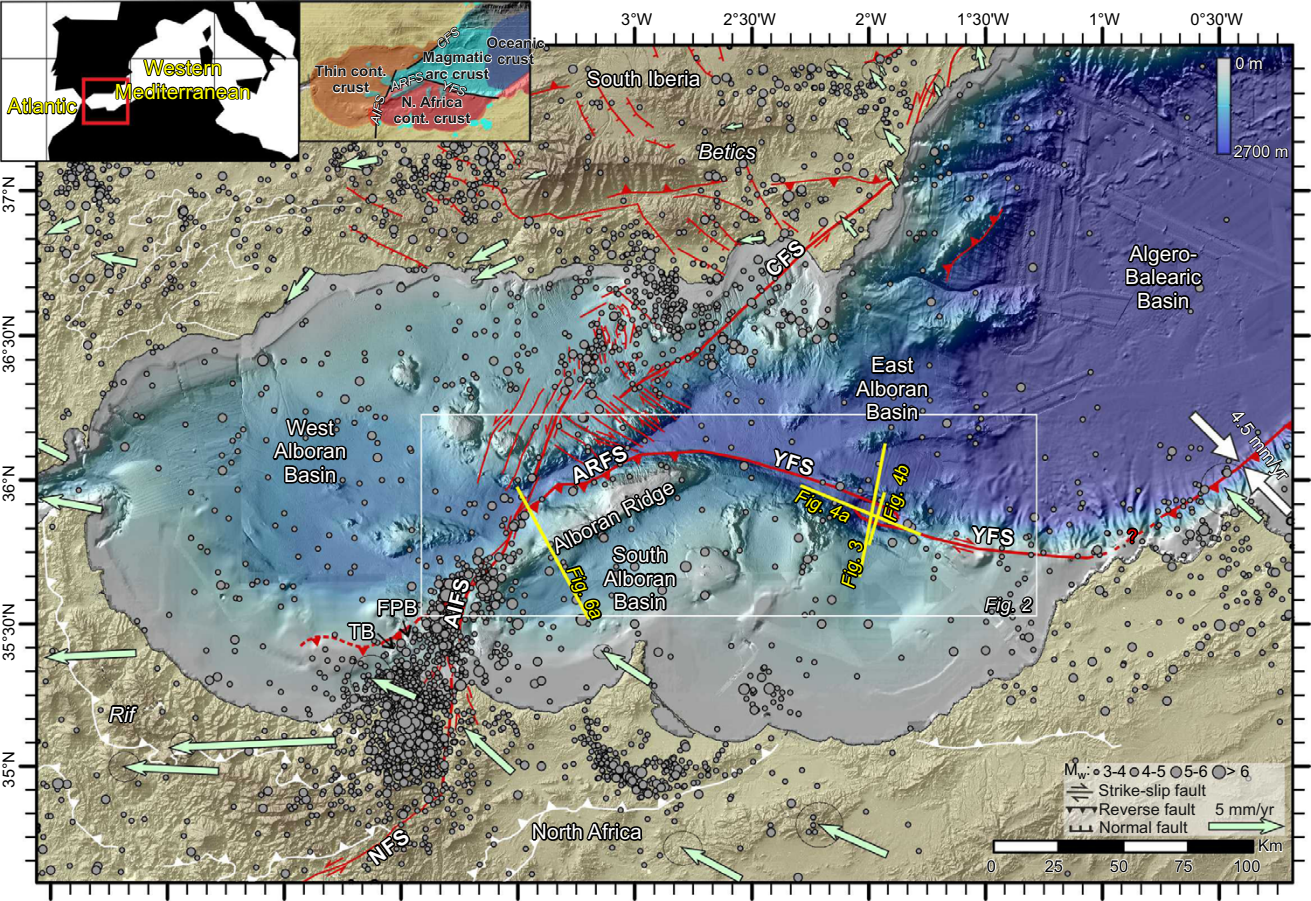
Bathymetric map of the westernmost Mediterranean. Tectonic structures, seismicity and GPS velocities in the region. Active tectonic structures are shown in red (modified after ref. 16), and the currently inactive tectonic structures related to the Miocene subduction process are shown in white (modified after ref. 6). The location of the crustal earthquakes (<30 km) that have occurred in this area since 1916 to today is shown (see legend for details, data from IGN catalogue, http://www.ign.es/). The GNSS velocities in a fixed Eurasian reference frame are shown by the green arrows, together with the ellipses for 95% confidence^11^. The regional convergence value determined from GPS measurements is shown by the white arrows^8^. Inset: Location and crustal domains of the study area (cont.: continental). AIFS: Al-Idrissi Fault System, ARFS: Alboran Ridge Fault System, CFS: Carboneras Fault System, FP: Francesc Pagès Bank, NFS: Nekor Fault System, TB: Tofiño Bank, YFS: Yusuf Fault System.

Rollback of subducting Tethys slabs drove the opening of the western Mediterranean basins^1^ until the latest Miocene, when extension stopped^2–4^. From the Pliocene to the Holocene, the region evolved into a contractional system driven by the 4.5 ± 1 mm/yr convergence between the Eurasia and African plates^5–8^. Crustal seismicity is abundant and scattered across a ~300-km wide region^9,10^ (Fig. 1), which GPS data indicate is deforming in a complex pattern of crustal blocks^11,12^ (Fig. 1). This broad region contains numerous active onshore and offshore faults^13,14^ (Fig. 1), which, with a few exceptions^15–17^, have undocumented slip histories. This distributed deformation has been interpreted as a diffuse Africa-Iberia plate boundary^10,11,18–20^. The moderate magnitude of instrumentally-recorded crustal seismicity^9,16,21^, is argued to further supports the diffuse-deformation model, with strain partitioned in medium size strike-slip and minor thrust and normal faults spread primarily across south Iberia and north Alboran (Fig. 1). However, the slow plate convergence (4.5 ± 1 mm/yr)^8^ might lead to large-earthquake cycles that are longer than instrumental records, resulting in undetected hazardous structures. The largest instrumentally recorded event is the M_w_ 6.4 Al-Idrissi earthquake^16^ but historical records of tsunamigenic earthquakes^22^ include the 1522 estimated M_w_ = 6.5 earthquake^23^, the 1680 estimated M_w_ = 7 earthquake^23,24^, and the 1804 estimated intensity 8 earthquake^25^; however, the submarine tectonic structures that caused these events remain undetermined.

**Fig. 2:**
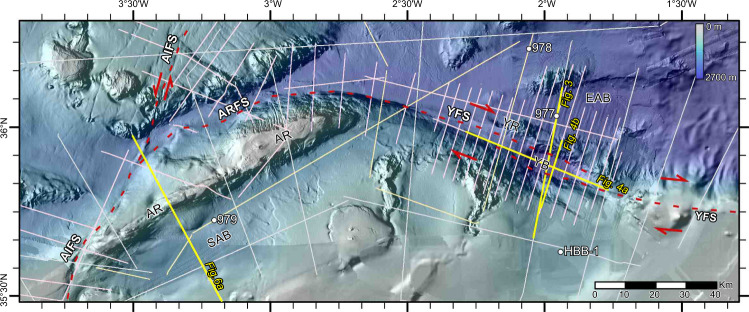
Detailed view of the studied area (see Fig. 1 for location). The trace of the main faults is shown by a discontinuous red line. The locations of the Multichannel Seismic profiles of TOPOMED (white lines), EVENT-DEEP leg 1 (pink lines) and EVENT-DEEP leg 2 (light yellow lines) are shown, as well as the commercial wells (HBB-1) and the ODP Leg 161 drill sites (white dots). AIFS: Al-Idrissi Fault System, AR: Alboran Ridge, ARFS: Alboran Ridge Fault System, CFS: Carboneras Fault System, EAB: East Alboran Basin, FP: Francesc Pagès Bank, SAB: South Alboran Basin, TB: Tofiño Bank, YB: Yusuf Basin, YFS: Yusuf Fault System, YR: Yusuf Ridge.

**Fig. 3:**
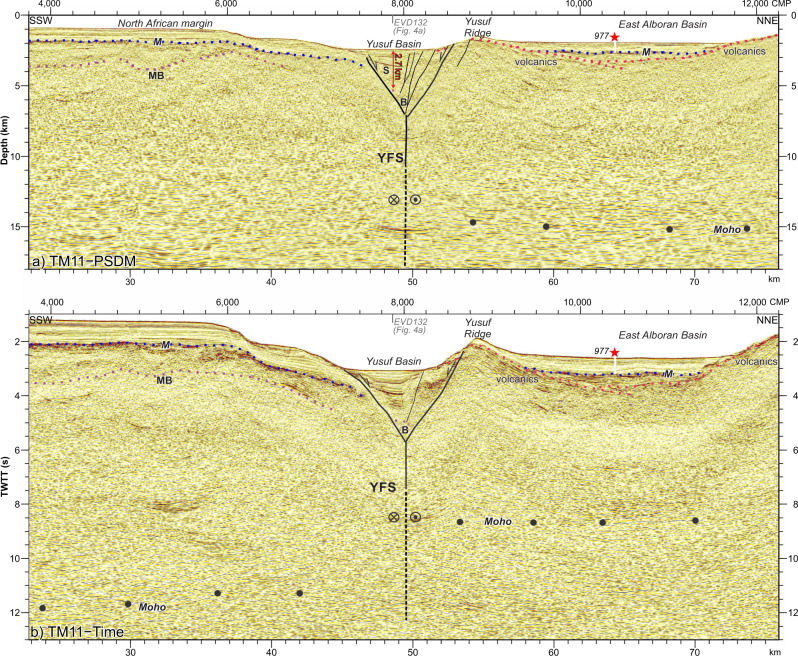
Deep structure of the Yusuf Fault System (YFS). **a** Pre-stack depth migrated section across the YFS (see Figs. 1 and 2 for location) showing the tectonic structure. The Yusuf pull-apart Basin (CMPs 7500–8500) has 2.7 km of maximum sediment thickness. **b** Time-migration of the same section. The Moho is located at ~11–12 s TWTT southern of the YFS, while it is located at ~8–9 s TWTT northern of the YFS. B: Basement, M: Messinian unconformity, MB: Metamorphic basement, S: Sediments, YFS: Yusuf Fault System.

Miocene geodynamic processes created three major crustal domains: thin continental crust flooring the West Alboran and Malaga sub-basins, magmatic arc basement under the East Alboran Basin, and oceanic crust under the Algero-Balearic Basin^2,3,26,27^ (inset Fig. 1). The South Alboran Basin is floored by the North-African continental crust^3,27^ (inset Fig. 1). These crustal domains boundaries are weak, inherited structures that have been re-activated during the Plio-Holocene contraction^3^. The largest identified features in the region are the Yusuf Fault System (YFS), the Alboran Ridge Fault System (ARFS), the Al-Idrissi Fault System and the Carboneras Fault System (Fig. 1). However, most kinematic models overlook these offshore structures^12,28^ that separate crustal blocks^29–31^. Previous studies of the slip of the Al-Idrissi^16^ and the Carboneras strike-slip fault systems^15^ conclude that they may have absorbed a small portion of the Plio-Holocene plate convergence (undetermined slip rates for the developing Al-Idrissi Fault System and between 0.8 and 1.3 mm/yr for the Carboneras Fault System)^15–17,30^. We here analyse the deformation structures associated with the YFS and ARFS. Focal mechanisms indicate that the YFS is a dominantly right-lateral strike-slip fault system and the ARFS produces right-lateral oblique thrusting^9,32^ (Fig. 1). These two features link laterally to form a ~300-km-long structure, with an associated seafloor relief larger than that of any other fault system in the region (Figs. 1, 2). The Plio-Holocene deformation has created ~2–3 km seafloor relief at the Alboran Ridge with the uplift gradually decreasing from the east, where uplifted volcanic basement crops out at the seafloor, to the west, where the ridge is narrower and formed by folded Neogene strata^4^ (Fig. 2). We analyse the deformation across the central segment of the ridge where the pre-stack depth migrated images display the real geometry of pre- and syn-tectonic sediment. The bathymetry shows that the frontal Alboran Ridge thrust fault is laterally kinematically linked to the Yusuf strike-slip system across a series of transpressive low ridges^3^. The YFS forms a pull-apart basin partially filled with ~2.7 km of mostly syn-tectonic sediments (Figs. 1, 2 and 3). The kinematics of these two fault systems are compatible with the Plio-Holocene contractional setting and with along-strike fault kinematics. Here we quantify the deformation absorbed independently by these major fault systems to determine their role in absorbing plate convergence and how they may contribute to regional seismic and tsunamigenic hazard. We present new reflection seismic images from four profiles that were acquired across the YFS and ARFS to characterize their structure, understand their kinematic evolution, and estimate their fault slip. Our results document that the YFS-ARFS fault system has accommodated ≥50% of the Plio-Holocene convergence, developing a discrete plate boundary fault system. This finding challenges the diffuse plate boundary model, and implies an underappreciated seismic and tsunami hazard in the region.

## Results and discussion

### The Yusuf Fault System

**Fig. 4:**
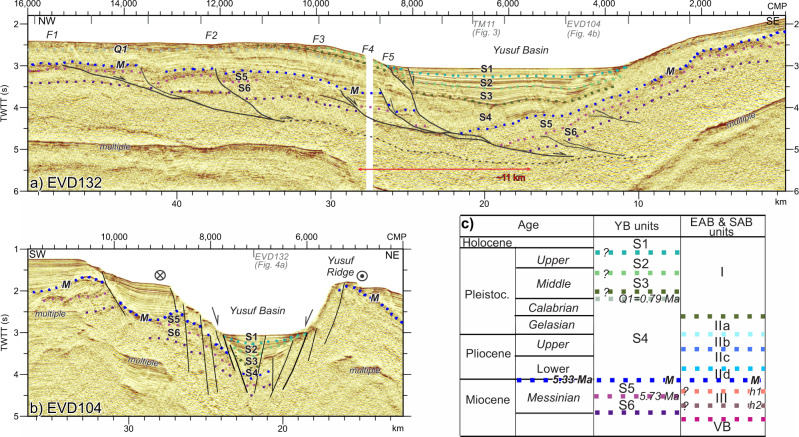
The Yusuf pull-apart Basin. **a** Time-migrated section crossing along the Yusuf pull-apart basin depocentre parallel to the YFS strike (Figs. 1 and 2; Vertical Exaggeration ~2.5). Pre-kinematic unit S6 is disrupted ~11 km below the basin depocentre (CMPs 9000–5000). **b** Time-migrated YFS-strike perpendicular section (Figs. 1 and 2; Vertical Exaggeration ~2.5). **c** Ages and seismostratigraphic units identified at the Yusuf pull-apart Basin (YB) and the East and South Alboran Basins (EAB and SAB, respectively).

The YFS is a ~160-km long dextral strike-slip fault system composed of two fault segments on its shallower part, that overlap forming the Yusuf pull-apart basin, and connect at depth into the same fault plane (Figs. 1, 2, 3 and 4). This fault system connects towards the east with structures absorbing the north-directed convergence between Africa and Eurasia (Fig. 1). We interpreted the structure of the Yusuf pull-apart basin using the seismostratigraphic units defined for the westernmost Mediterranean^4^ calibrated with the ODP Leg 161 and industry wells^33,34^ (Fig. 2, see ‘Methods’). The first YFS activity inferred from the oldest syn-tectonic deposits in the pull-apart basin is late Miocene to early Pliocene (Fig. 4). Different intra-basement reflections occur on either side of the YFS^3,27^ (Fig. 3). North of the YFS, Moho reflections at ~15-km depth delineate a <14-km-thick crust under the East Alboran Basin (~6 s TWTT thick crust, with the Moho located at ~8.5 s, Fig. 3a, b CMPs 8000–12,000), similar to wide-angle seismic measurements nearby^26^. In contrast, south of the YFS, abundant intra-crustal reflectivity is underlain by faint Moho reflections at ~18–20-km depth^3,27^ (~11–12 s TWTT, Fig. 3b CMPs 4000–7000), which deepen to ~30 km near the North African coast^3,27^. The abrupt change in crustal thickness across the YFS indicates that the YFS is a lithospheric scale tectonic boundary.

From younger to older, the pull-apart basin contains six sedimentary units (S1–S6, Fig. 4a–c). Their age was calibrated by correlation with the sediment units from the East and South Alboran Basins following two time markers: the Q1 horizon (0.79 Ma) and the M horizon (5.3 Ma) (Fig. 4c), drilled on ODP-161 sites 977 and 978^33^ and the HBB well^34^ (Figs. 2 and 3). In the YFS area, the early Messinian unit S6 is faulted (Fig. 4a CMPs 9000–5500), but the segments display roughly constant thickness, which supports its pre-kinematic deposit (Fig. 4a CMPs 16,000–10,000). In contrast, the tilt of strata and thickening of units S1 to S5 towards the pull-apart depocentre (Fig. 4a CMPs 10,000–3000, Fig. 4b CMPs 8000–5000), indicate that the late Messinian and Plio-Holocene infill is syn-kinematic with the opening of the pull-apart basin. The largest thickening towards the pull-apart depocentre occurs for unit S4, supporting that the main phase of extension occurred during the Pliocene–early Pleistocene.

**Fig. 5:**
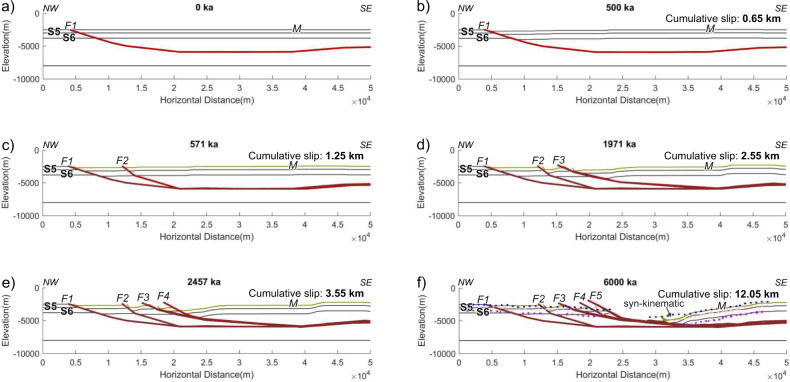
Evolution of the Yusuf Fault System (YFS). **a**–**e** Results of the forward models for the YFS. Years of activity for each model step are shown in the upper part of each panel. Faults F1 to F5 are progressively activated. Inactive faults are shown in dark red, and the active fault in bright red. The total slip accommodated by the fault system in this model is 12.05 km. **f** The final stage of the model (6 Ma) is compared with the depth-converted horizons of profile EVD132 (Fig. 4a), which runs parallel to the YFS along the pull-apart basin depocentre.

Earthquake focal mechanisms indicate that the YFS is dominantly a dextral strike-slip system^32^, so that the ~11 km gap of the base reflection of the pre-kinematic unit S6 parallel to the fault strike represents the minimum cumulative slip on the normal faults controlling extension within the pull-apart since 5.73 Ma (Fig. 4a CMPs 9000–5500). To further constrain total slip, we have modelled the main extensional faults creating the pull-apart (faults F1–F5 in Fig. 4a) using the fbfFOR software^35^ (Fig. 5, see ‘Methods’ for details). The software allows one active fault at a time, and we sequentially modelled F1 to F5 (Figs. 4a and 5). Some faulting may have overlapped in time, but we estimated the cumulative extension using the pre-kinematic units (considering the late Messinian unit S5 as pre-kinematic). The modelling estimates a total slip accommodated by F1–F5 faults of ~12 km, with ~3.5 km during F1–F4 and ~8.5 km during F5 (Fig. 5). This estimate agrees with the ~11 km gap of unit S6.

The contribution of faults with offset smaller than seismic resolution is unconstrained, although as much as 25–60% of the total extension has been estimated for extensional settings^36^. Although speculative, that could increase total slip to 16–30 km for the YFS. Numerical models of pull-apart basins support that the 3 km subsidence of Yusuf pull-apart might require 20–30 km of slip^37^. Thus, the YFS accommodates a minimum 12 km slip, but it might be considerably larger. A better slip estimation would require to obtain both higher resolution seismic data and 3D numerical modelling for understanding pull-apart basin formation.

### The Alboran Ridge Fault System

**Fig. 6:**
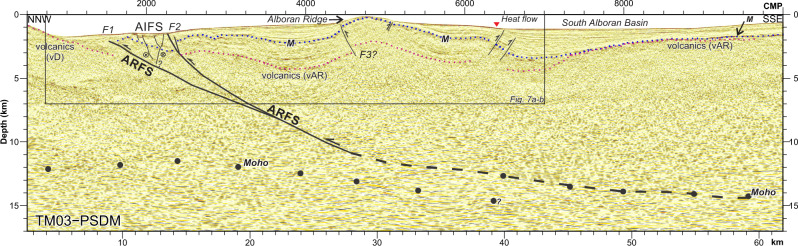
The Alboran Ridge Fault System (ARFS) depth structure and evolution models. Pre-stack depth migrated section across the Alboran Ridge (see Figs. 1 and 2 for location). The crustal dimension of the ARFS is inferred for the different reflectivity observed on the two sides of the fault, together with the different Moho depths (black dots). AIFS: Al-Idrissi Fault System, M: Messinian unconformity, vD: volcanics Djibouti Plateau, vAR: volcanics Alboran Ridge.

**Fig. 7:**
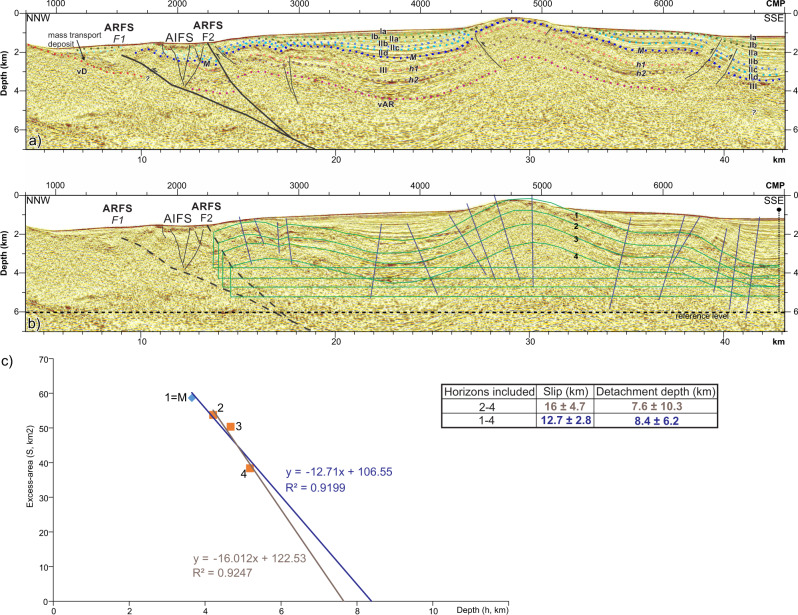
Excess-Area method. **a** Detail of the shallow structure of the Alboran Ridge (see Fig. 6a for location). The seismostratigraphic units are the same as those for the East Alboran Basin (Fig. 4c). vD: volcanic basement at the Djibouti Plateau, vAR: volcanic basement at the Alboran Ridge area. **b** Same section as Fig. 7a. The different horizons used in the Excess-Area method are shown in green, and the main axial planes of the folds are shown in dark blue. **c** Excess-Area results. Two results are shown: In blue, the linear regression including the Messinian horizon (labelled as 1); and in brown, the linear regression without including the Messinian horizon.

The ARFS is the largest tectonic structure in the region, with up to 2 km of seafloor relief along its 130 km of length (Figs. 6 and 7). The bathymetry shows the lateral connection of the ARFS and the YFS (Figs. 1 and 2). To the west, the Alboran Ridge is separated from the Francesc Pagès and Tofiño banks by the Al-Idrissi Fault System^16^ (Figs. 1 and 2). The ARFS separates different crustal thickness on either side^3^ (inset Figs. 1 and 6). To the north of the ARFS, the Moho is delineated by discontinuous reflections between ~11–13-km depth, defining a <10 km thick crust (Fig. 6 CMPs 1500–4000). To the south of the ARFS, the Moho deepens to ~15 km, defining a crust >13 km thick (Fig. 6 CMPs 5000–10,000). The volcanic basement is different to the north and south of the ARFS-F1^3,4^ (indicated by vD and vAR in Figs. 6 and 7a). The Plio-Holocene sedimentary units are the same age as those in the East Alboran Basin (Fig. 4c), with the addition of two Late Miocene horizons (h1 and h2, Fig. 7a) of imprecisely known age (Messinian?). A well-established stratigraphy, growth strata, and layer tilting support an earliest Pliocene age for the initiation of shortening deformation^4^ (Figs. 4c and 7a). Shallow deformation indicates a frontal thrust and a main splay (ARFS-F1 and ARFS-F2, Figs. 6 and 7). ARFS-F1 was probably active since early Pliocene (units IId-c), although recent deformation by Al-Idrissi fault system obscures the interpretation (Figs. 6 and 7a CMPs ~2000). The Messinian unit (III at Fig. 7) shows internal parallel reflections, supporting its pre-kinematic character (Fig. 7a CMPs 4000–6000). It has an erosive top (marked as “M” in the figures). The wedging of units II and I, thinning towards the Alboran Ridge high (Fig. 7 CMPs 4000–6000), supports that the ARFS-F2 activity initiated in the early Pliocene and remains active (Fig. 7a).

To quantify total slip, we used the fbfFOR^35^ software to test whether a fault-bend-fold geometry explains the Alboran Ridge deformation (see ‘Methods’). We tested different fault geometries, including a main thrust and a splay, and different initial configurations. The results support a detachment level at ~15-km depth and a total slip between 10 and 12 km, mainly accommodated by a detachment reaching the surface at the front of the Alboran Ridge (Supplementary Fig. 1). However, none of the tested geometries fully explains the configuration observed in the seismic images (Supplementary Fig. 1c) because they do not (1) reproduce the whole deformation of the layers (e.g., Fig. 7a CMPs 2750–3000, Supplementary Fig. 1) and (2) cannot properly include the secondary faults, accommodating a relatively minor part of the deformation (Fig. 7a CMPs 4500 or 6500). The modelling approach imposes first-order faults, cutting the entire sequence and the seafloor, and only one active fault at each time step. The data shows faulting at different scales, with not all the faults reaching the surface (e.g., blind thrust, Fig. 7a CMPs ~1500 and 4500), and probably with similar ages of activity, that cannot readily be included in the model.

Due to the forward modelling restoration limitations, we used area-balance methods to calculate the slip necessary to generate the relief of the Alboran Ridge. We focused on the slip accommodated by ARFS-F2 due to the erosive levels and later deformation affecting the sediments north of this fault, which obscures the interpretation of the layers (Fig. 7a). We estimated the slip on ARFS-F2 using the Excess-Area method^38^ (Fig. 7, see ‘Methods’). The inputs for the method are: (1) The deformed area (S) for each pre-kinematic unit considered; and (2) The depth (h) of the top of these units. The slope of the regression line fitting these points delimits the total slip, and the point for S = 0 determines the detachment depth. Four pre-kinematic reflections identified in the PSDM images cross over the ARFS, including the M reflection (Fig. 7b). However, the M reflection measurements have a higher level of uncertainty due to the erosion that may have affected late Messinian sediments in this region^4^ (Fig. 7a). To fulfil the requirements of the excess-area methodology, we restored the geometry of the layers where they are eroded following the axial planes of the fold (Fig. 7b). Including the M horizon measurements, the estimated slip is 12.7 ± 2.8 km; and excluding the M reflection measurements the estimated slip increases to 16 ± 4.7 km (Fig. 7c). Although the area measures have considerable uncertainty associated (see ‘Methods’), slip values >10 km are supported by the forward modelling results. The estimated fault detachment depth using the Excess-Area method is ~7.5–8.5 km with the high ± >6 km uncertainty intrinsic to the Excess Area method^39^.

**Fig. 8:**
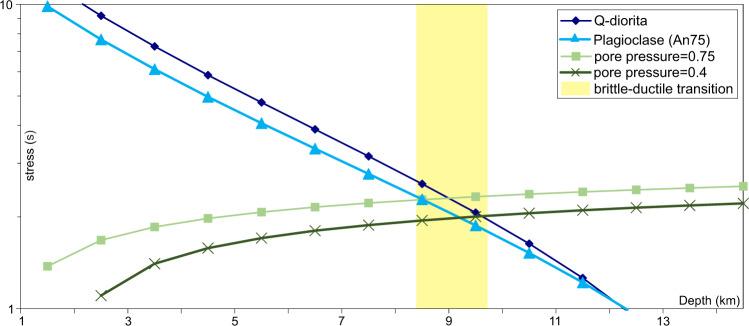
Brittle–ductile transition estimation from a differential stress (*σ*) versus depth plot. In blue is depicted the resistance curves for a Quartz-diorite and a Plagioclase (An_75_), in agreement with the composition of the rocks sampled in the area. In green, we show the frictional stress results obtained by applying the Anderson equation for two different pore pressures: *λ*_1_ = 0.4 and *λ*_2_ = 0.75. The results support a brittle–ductile transition located at ~8.5–10-km depth.

We estimated the brittle–ductile transition depth based on the geothermal gradient and the rheological behaviour of the rocks sampled. We followed the relationship proposed by Ranalli and Murphy^40^ to perform the geothermal gradient estimations (Eq. 1 in ‘Methods’). The input parameters were taken from the measurements of Polyak et al.^41^. The brittle–ductile transition depth has been estimated by the comparison of the stress obtained applying the dislocation creep^42^ (Eq. 2 in ‘Methods’) with the frictional stress obtained by the Anderson equation (Eq. 3 in ‘Methods’). Results are shown in Fig. 8, and support that the brittle–ductile transition in the area is located between ~8.5- and 10-km depth below the southern flank of the Alboran Ridge (Figs. 6 and 8). Previous studies of the thermal structure of the area support that the thermal gradient is lower towards the basin margins^43^, and thus, the brittle–ductile transition should be deeper towards the south. The brittle–ductile depth is slightly deeper than the depth obtained with the Excess-Area method, although within its error bar. Being the ARFS a first-order tectonic structure deforming >10 km of crust, and based on the similar values obtained for the detachment depth and the brittle–ductile transition, we suggest that the detachment may be rooted at the brittle–ductile transition. In addition, the resulting detachment depth values are consistent with the location of the change in basement reflectivity observed in the seismic profiles (Fig. 6). Thus, due to the different types of crust observed on the two sides of the ARFS (inset Fig. 1) and the depth suggested by the forward modelling and the excess-area methods (Fig. 7c) we propose that the ARFS decollement is rooted at Moho level at 10–14-km depth (Fig. 6).

To obtain further constraints on the total slip by another independent method, we applied the equations derived from kinematics analysis of fault-related folding^44,45^ (Supplementary Fig. 2). In particular, these equations relate uplift to slip along a fault through the detachment angle (see ‘Methods’). We considered that most of the slip is accommodated by the main detachment fault, as supported by the forward models (Supplementary Fig. 1). On the seismic images we measured a ~2 km uplift and assumed a low angle for the basal detachment thrust, which is consistent with this type of structures and with the forward models results (Fig. 6, Supplementary Figs. 1, 2). Because the fault-related folding method depends on the interpreted geometry of the detachment and the exact depth of the fault cannot be well constrained, a high level of uncertainty is associated with these results. Assuming a detachment angle of 5° the result gives ~23 km of slip, and assuming an angle of 10° the result gives 11.5 km of slip (Supplementary Fig. 2). The resulting ~11.5–23 km of slip is in reasonable agreement with the Excess Area results (16 ± 4.7 km) and the total slip obtained for the complementary YFS (minimum 12 km, 16–30 km including sub-seismic deformation).

The methods we have used have an uncertainty that is difficult to quantify, partially because the deep fault trace has not yet been clearly identified on seismic images. It is however significant that all methods estimate similar slip values and a detachment level in the lower crust. In addition, all estimations consider plane strain which is consistent with the Alboran Ridge trend in relation to the current NW-SE plate convergence (Fig. 1). However, transpressive focal mechanisms^16,21,46^ are also observed in the area. If the Alboran Ridge is the result of transpression instead of plane strain, the total slip value accommodated by this structure will be larger, as these methods only estimate the 2D shortening parallel to the line of section.

### The plate boundary fault system

**Fig. 9:**
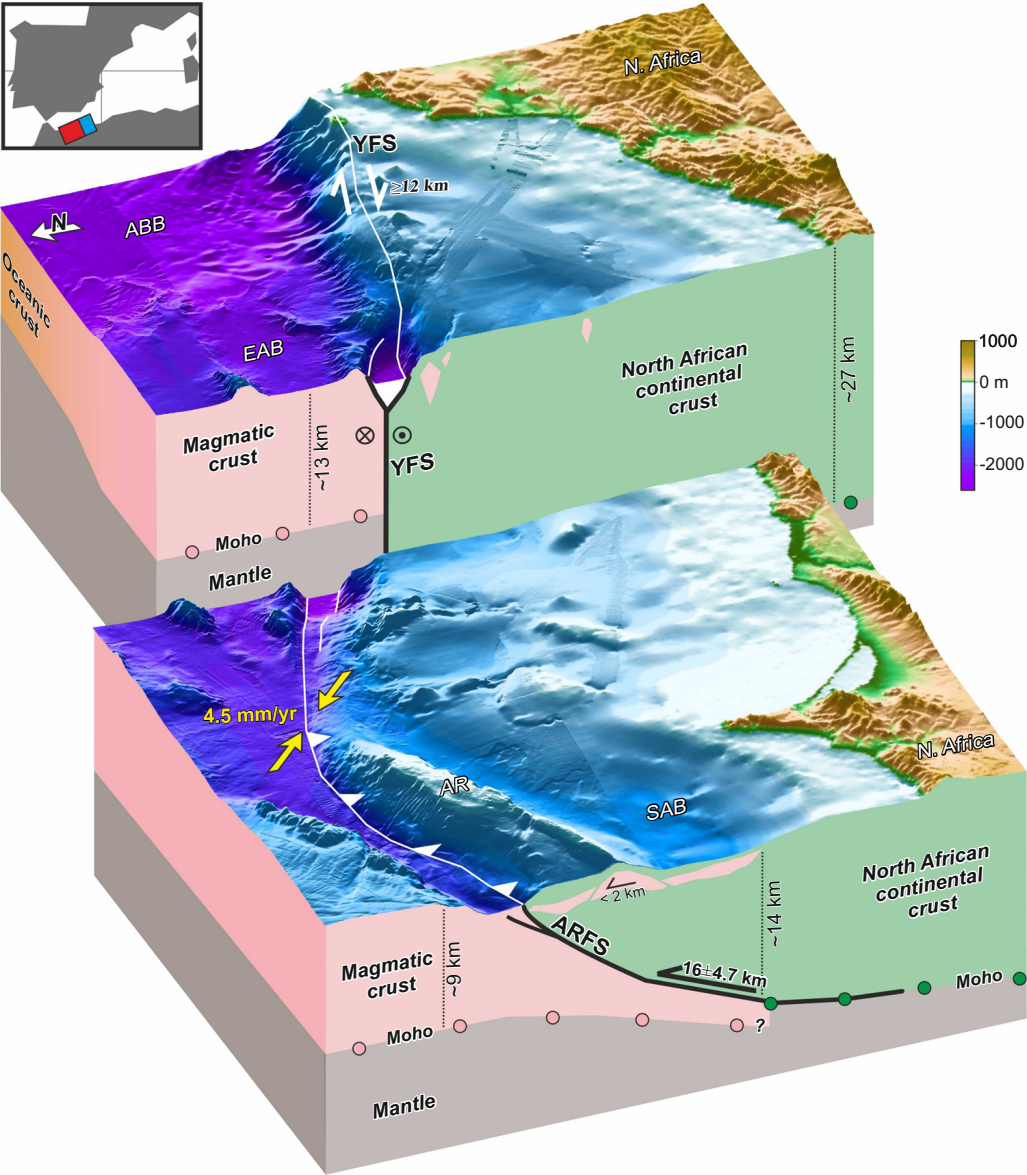
Three-dimensional view showing the surface and the deep structure of the African-Eurasian plate boundary in the western Mediterranean (point of view located at N290° and elevated 30°). The deep structure is interpreted based on the depth-migrated multichannel seismic sections (vertical axis not to scale). The Plio-Holocene slip values associated with the Yusuf and Alboran Ridge fault systems are shown, as well as the regional plate convergence values (yellow arrows). Volcanic intrusions in the North African continental crust are shown as red polygons. Inset: location of the 3D views shown. AR: Alboran Ridge, ARFS: Alboran Ridge Fault System, EAB: East Alboran Basin, SAB: South Alboran Basin, YFS: Yusuf Fault System.

The estimated total slips of minimum 12 km for the YFS (from 16 and up to 30 km including the sub-seismic deformation) and 16 ± 4.7 km for the ARFS have large uncertainties intrinsic to the available data and existing methods of analysis for thick skin tectonics. However, the total slip calculated with the different methods for the two systems is consistent. Furthermore, total slip estimations are of the same magnitude for the YFS and ARFS, which further supports that the two systems may be laterally linked and form a ~300-km-long kinematically-compatible single system as depicted in Figs. 1 and 9.

The YFS and ARFS initiated their current kinematics during the late Messinian–early Pliocene regional geodynamics change. The orientation of the paleo-stresses during the Plio-Holocene^6^ is consistent with a constant Eurasia-Africa NW-SE plate convergence from ~8 Ma^47^. Assuming the current 4.5 ± 1 mm/yr convergence rate^8^, the convergence between Iberia and Africa during the Plio-Holocene (<5.3 Ma) would have been 24 ± 5 km. Thus, at least half of the Plio-Holocene plate convergence have been absorbed by the YFS-ARFS fault system as the minimum estimate is 12 km of slip, but could be closer to total plate convergence given the uncertainty in total deformation observed by the fault systems.

Although numerous other faults occur in the region (Fig. 1), it is unlikely that a large structure that could accommodate significant deformation has remained undetected to the numerous dedicated studies. The two other major known structures in the region, the Al-Idrissi and the Carboneras Fault Systems are well studied, and have not accommodated significant slip (Fig. 1). The Al-Idrissi Fault System shows minor Plio-Holocene deformation^16^. It is made up of several segments that are currently developing and it is unclear whether they are yet interconnected^16^. The estimated slip rate of the Carboneras Fault System is 0.8–1.3 mm/yr^15,17,30^. North of the Carboneras Fault System, the onshore Eastern Betics contains numerous active faults that currently accommodate a small part of the convergence. These are relatively short structures associated with NNW-SSE shortening values of 0.6 ± 0.2 mm/yr^17^. Previous studies support that the amount of slip cumulatively accommodated by the distributed secondary fault systems is comparatively minor^16,17,30^, can be considered intraplate and absorbs much less deformation that the slip accommodated by the YFS-ARFS system, as estimated in our work. Shortening rates of 0.6 mm/yr are translated in ~3 km of total slip during the Plio-Holocene, and thus, represent only ~12% of the total plate convergence, while the >12 km measured for the YFS-ARFS represent ≥50% of the plate convergence. Although the YFS-ARFS plate boundary is not as mature as in a well-established subduction or collision system, it appears clear that other faults in the region absorb a comparatively minor part of the convergence. In addition to the secondary faults accommodating the convergence, normal faults in the central and western Betics accomodate 3–4 mm/yr of NE-SW extension but these processes appear to be triggered by tearing of the underlying subducted slab and not directly by the current plate convergence^48–51^.

The YFS-ARFS fault system has developed along the contact zone of pre-existing crustal domains of continental and magmatic arc types of crust^3,27^ (inset Figs. 1 and 9). The inherited structure of the domain boundary may have facilitated focusing the deformation. Inherited structures may also govern the development of the plate boundary in the Gulf of Cadiz in the Atlantic^52,53^. The 16 ± 4.7 km total slip of the ARFS has occurred along a decollement where the North African continental crust thrusts over a thinner magmatic arc crust (Fig. 9). Although subduction initiation has been speculated for the north Algerian margin^54^ and as a future scenario for the Gulf of Cadiz^52^, it is unlikely that this scenario could occur here, because the lithosphere in the region is young and comparatively buoyant^55^. Towards the east, the YFS-ARFS linked fault system connects with the tectonic structures interpreted to reactivate the north Algerian margin^56,57^ (Fig. 1), although the deformation accommodated by these structures has not been yet quantified.

The current conventional wisdom for this region is that the slow convergence rate and distributed deformation implies a moderate seismic and tsunami hazard^22,58–60^. However, the YFS and the ARFS are crustal-scale structures that are favourably oriented for absorbing most of the current plate convergence. Both, historical seismicity and tsunamis in the Alboran region, support the occurrence of earthquakes larger than those instrumentally recorded. The active ARFS and YFS are structures >300-km long, with a brittle–ductile transition at 8.5–10-km depth and thus have the potential to create a large earthquake with a thrust slip component in the ARFS. Earthquake fault-scaling relations for continental regions^61^ estimate a maximum earthquake with a M_w_ = 7.4 for the YFS and M_w_ = 8 for the ARFS. If both systems rupture together, the estimated maximum earthquake has a M_w_ = 7.7 considering a strike-slip kinematics and M_w_ = 8.8 considering a reverse fault kinematics (Supplementary Table 1). These structures have a poorly-documented seismic record and possibly long recurrence periods, and they are typically not included in the seismic hazards maps^62^. Hence, the ARFS and YFS need to be integrated in probabilistic seismic hazards analysis to realistically characterize their seismogenic and tsunamigenic potential^63^, which is not yet taken into account^58^.

Our quantitative estimations support the ongoing development of a well-defined plate boundary fault that absorbs most of the convergence between Africa and Iberia (Fig. 9). Therefore, the commonly assumed diffuse-deformation plate model does not adequately describe the observed tectonic structures. The tectonic role of the YFS-ARFS needs to be integrated into kinematics models of the western Mediterranean, and its associated earthquake cycle should be characterized, as these faults have the potential to produce large to perhaps great earthquakes. Therefore, the YFS-ARFS should be considered in earthquake hazard analyses for assessing seismic and tsunami risk in the region.

## Methods

### Seismic data acquisition and processing

The seismic data presented in this work is based on two different datasets, the TOPOMED and the EVENT-DEEP Leg 1 seismic profiles (Fig. 2). The TOPOMED multichannel seismic data were acquired on September–October 2011 on board the Spanish RV “*Sarmiento de Gamboa*”. During this cruise, deep-penetration seismic data were acquired to image the Alboran Basin at a crustal scale. A *Sercel* solid-state multichannel digital streamer (408–480 active channels separated by 12.5 m) up to 6-km long and two large volume G-II airguns were used. The EVENT-DEEP Leg 1 was designed to obtain a high-resolution image of specific structures of the basin, including the Yusuf Fault area. This cruise took place during 2010, also on board RV “*Sarmiento de Gamboa*”. In order to study the most recent sedimentation and active faults, a 600 m Sercel SEAL multichannel seismic streamer of 600 m (96 channels, separated 6.25 m) was rented from the Exploration-Electronics Company, and a G-gun II airgun array was fired at 800 c.i.

The TOPOMED profiles were processed in two domains: time and depth. The time domain processing flow included (1) minimum-phase conversion, (2) streamer geometry definition accounting for feathering, (3) common mid-point sorting, (4) spherical divergence correction, (5) predictive deconvolution in the Tau-P domain (to eliminate the bubble and short-period multiple reverberations), (6) surface-consistent deconvolution, (7) Surface Related Multiple Elimination (SRME) and (8) Radon filter multiple attenuation. Depth domain processing flow input is the final output of the processing flow described above, and included: (1) a deconvolution designed to remove the dipping noise and amplitude spikes; (2) an accuracy velocity analysis based on the residual analysis of the Common Image Gathers until the final velocity model was obtained in the depth-domain; (3) the stacking of the final Pre-Stack Depth Migrated (PSDM) section at different offsets, including an external mute, which makes it possible to delete the wavelet stretching in far offsets; (4) a Trimmed Mean Dynamic Dip Filter to remove high-amplitude noise and locally weak coherent events; (5) a FX deconvolution to laterally enhance the signal; and (6) a depth and spatial variant band-pass filter to filter undesired remaining frequencies and that follows the same criteria as in the time domain filtering.

The EVENT-DEEP Leg 1 profile processing flow in the time domain included: (1) static correction and quality control, (2) filtering, including bandpass and FK filtering, (3) spherical divergence correction, (4) velocity analysis and stack, (5) post-stack migration and (6) amplitude equalization.

### Seismic data interpretation

The stratigraphic units have been interpreted based on the seismostratigraphy proposed for the entire area in Gómez de la Peña et al.^4^. This seismostratigraphy is done based on the correlation with previous seismic studies, with ODP leg 161 sites^33^ and industry wells^34^ (Figs. 1 and 2). The Moho is interpreted following previous studies of the seismic character of the crust, that defined the Moho location on multichannel seismic data based on comparison with wide-angle seismic data. These studies support that on multichannel seismic sections, the Moho can be imaged as a bright reflection but also as the reflections at the base of the lower crust reflectivity (ref. 3 and references therein).

### Slip estimation

#### Forward modelling

We attempt to quantify the deformation by reproducing it using a forward modelling software, in particular, the fbfFOR software^35^. In this software, you defined (1) the initial configuration of the layers (pre- and syn-kinematic), (2) the active fault geometry, (3) the slip rate and (4) the timing of the deformation. Initial models and results of the forward modelling are shown in Figs. 5 and 6b, c, for the YFS and the ARFS, respectively. The main issues that we found when modelling the observed structures were related with the impossibility to include (1) minor faults into the model and (2) contemporary active faults. We obtained a model that reasonable fits with the YFS current configuration, giving as a result 12.05 km of slip accommodated by the main faults observed in the section (Fig. 6). Results of the forward modelling have been compared with the time-depth converted horizons of profile EVD132. Based on previous studies of fault systems, the large faults individually accommodate 40–75% of the total extension, while the small faults accommodate the remaining 25–60%^36^. Thus, the total extension along the YFS is 16–30 km. In the case of the ARFS, a fault-bend-fault model cannot fully explain the observed deformation (Fig. 7b, c). We infer that these issues are related to modelling limitations: (1) the assumption that all the hanging wall rock has the same rheology, when it is formed by at least three different layers—the sedimentary cover, underlying volcanic/igneous basement and possibly deeper intruded continental crust; (2) the impossibility of model secondary faults and first-order faults active at the same time; and (3) the method limitations for modelling thick-skin tectonics. Due to the lack of constraints in the sedimentation and compactation rates and the impossibility of model more than one active structure at the same time, we exclude the syn-kinematic sediments from the forward modelling.

#### Strike-slip quantification

We used the results from the mathematical modelling performed by Rodgers^37^ based on the elastic dislocation theory to quantify the offset along the master faults of the Yusuf Fault system. An isotropic, homogeneous and linear half-space is assumed. We chose this particular approach because is a simple 2D approach to evaluate first-order structures. The results show that the geometry of the pull-apart basin is controlled by the amount of overlap and the separation between the two fault segments, and whether the faults cut the seafloor or not. The Rodgers^37^ model supports that the depth of the basin depocentre is ~10–15% of the total offset accommodated along the fault. This percentage depend on the amount of overlap in relation with the separation of the fault segments, being 10% when the overlap is twice the separation and 15% when there is no overlap, although it is also dependent on the elastic properties of the area, which are not considered in this approach. The maximum depth in the pull-apart is measured along a line joining the ends of the two master faults. The location of profile TM11 is in accordance with this assumption (see Fig. 1), so we measured the depth of the pull-apart along this section (Fig. 3a). The maximum syn-tectonic sediments thickness measured in the pull-apart on the TM11 PSDM section is 2.7 km, although the pull-apart has not full colmatation (Fig. 4a). Thus, the accommodation space created by the pull-apart opening is ~3 km (Fig. 4a). Following the estimation determined with the Rodgers^37^ method, the theoretical offset of the YFS is between the 10–15% of the maximum 3-km depth measured at the Yusuf pull-apart, and thus, between 20 and 30 km. The relations obtained by Rodgers are only valid to make first-order predictions, but not to explain the detail of the structures.

#### Excess-Area method

The excess-area graphical technique^38^ uses the excess-area to determine the depth to the detachment and the slip along it. The excess-area of a fold is the area of material uplifted by deformation^38^. The excess-area graph is generated by plotting the excess areas (S, Fig. 7b, c) of different stratigraphic levels versus the depth of these levels to a constant reference horizon (h, Fig. 7b, c). The result is a straight line for detachment folds (Fig. 7c), where the slope is the displacement on the detachment and the value S = 0 is the detachment depth. To use the excess-area method, the horizon relief must be a consequence of the measured slip. For syn-tectonic deposits and sedimentary layers that have a stratigraphic relief, it is necessary to restore the original geometry in order to perform accurate measurements^64,65^.

We used four pre-kinematic horizons, each of a constant age across the Alboran Ridge to estimate the slip values in the Alboran Ridge area and fulfil the methodological requirements (Fig. 7b). Measurements were carried out at a 1:1 scale plot of the Pre-Stack Depth Migration of profile TM03. Due to the acquisition geometry of the seismic profiles, using a 5100 m-length streamer, the depth section provides an accurate geometry until at least 4–5-km depth. Thus, all mapped horizons used in the Excess-Area methods are shallower than 4-km depth (Fig. 7b). There are possible errors in the measurements due to (i) non-parallel stratification of all sedimentary layers, (ii) unknown geometry prior to the shortening, (iii) uncertainty in the area measurements due to later deformation that obscures the interpretation, and (iv) possible erosion of surfaces. Therefore, to minimise these uncertainties, we defined constant thickness layers (especially where erosive surfaces are found), following the axial planes of the folding (Fig. 7b). The regression coefficient (R) of determination is R^2^ > 0.9. Based on the minimum mean square estimation, the total error is estimated as <25% of the calculated slip. The average error for the detachment depth estimation based on a compilation of the results^39^ is >45%. Thus, we are aware of the high uncertainty associated with the detachment depth. However, based on heat-flow measurements and the rheology of the basement rocks in this area, similar depths have been obtained for the brittle–ductile transition, which supports the validity of our results. In addition, these results are broadly consistent with the reflectivity observed in the multichannel seismic sections.

#### Fault-related folding method

The fault-related folding model^44,45^ proposes that the total uplifted area (*u*) is related to the slip along the detachment (*d*) and the detachment angle (*θ*), following the relation *d* = *u*/sin *θ* on a mature fault-bend-fold where layers thickness and length are preserved (Supplementary Fig. 2). We measured the maximum uplift on the PSDM section (Fig. 6a and Supplementary Fig. 2) and we assumed a low angle detachment, consistent with this type of structure and with the detachment geometry obtained on the forward models (Supplementary Fig. 1). We measured a maximum uplift equal to 2 km (*u* in Supplementary Fig. 2b), and assumed a detachment angle between 5° and 10° (Supplementary Fig. 2a). For a 5° angle detachment the resulting slip is 22.9 km (*d* = 2/sin 5°), and for a 10° angle is 11.5 km (*d* = 2/sin 10°).

### Brittle–ductile transition

*Estimation of the geothermal gradient*^40^ (Eq. 1):

1 $$T={T}_{0}\,+\,\frac{\left({J}_{{qo}}-{J}_{{qr}}\right)d}{K}\,\cdot \,\left(1-{e}^{\frac{z}{d}}+\frac{{J}_{{qr}}}{K}\,z\right)$$

Where *T* is the resulting temperature, *T*_0_ the temperature at the surface, *J*_qo_ the initial thermal flow, *J*_qr_ the reduced thermal flow, *d* the depth where *A* = *A*_0_e^−1^, being *A* the radiogenic heat production, *K* the thermal conductivity and *Z* the depth. The heat flow measured on the sea-floor surface in the South Alboran Basin has a mean value of 121 mW/m^2^, and the *T*_0_ 285 K^41^. This high-value is related with the late Miocene–early Pliocene volcanic activity in this area. For the thermal conductivity we used a mean value for a diorite, 2200 mW/mK, and for the radiogenic heat production, 0.002 mW/m^3^, as described onshore in the Betics.

*Dislocation creep*^42^ (Eq. 2):

2 $$\sigma ={\left(\left(\frac{\varepsilon }{{A}_{D}}\right)\right)}^{1/n}{\exp }\cdot \left(\frac{E}{{nRT}}\right)$$

Where *σ* is the stress difference, *R* the gas constant, *T* the obtained temperature in Eq. 1 (depth dependent) and *A*_D_, *n* and *E* are the creep parameters, which depend on the composition. We tested two different compositions, using the creep parameters for Q-diorite and for plagioclase (An_75_) (Fig. 8).

*Anderson equation* (Eq. 3):

3 $$\sigma =\alpha \rho {gz}\cdot \left(1-\lambda \right)$$

Where *ρ*, *g*, *z* and *λ* are referred to the density, gravity, depth and pore fluid factor, and *α* is a numerical parameter that depends on the type of fault (3 for thrust, 1.2 for strike-slip and 0.75 for normal faults). Due to the geological framework, we used *α* = 1.2 (as most of the focal mechanism in the area show strike-slip component), *ρ* = 2.7 kg/m^3^ and we used two different values for the pore pressure, *λ*_1_ = 0.4 and *λ*_2_ = 0.75 (Fig. 8).

## Supplementary information

Supplementary Information

## Data Availability

Bathymetric and topographic data used in Figs. 1, 2 and 4 are our compilation of the publicly available (EMODnet Bathymetry^66^ and NASA SRTM topography^67^) and bathymetry collected in SARAS cruise^68^. Seismic data plotted in Fig. 1 are from the *Instituto Geográfico Nacional* (IGN, http://www.ign.es/web/en/ign/portal/sis-catalogo-terremotos) and GNNS velocity vectors and associate errors are from ref. 11 Seismic data were acquired during the Barcelona-CSI marine cruises TOPOMED-GASSIS and EVENT-DEEP Leg 1 (http://gma.icm.csic.es/sites/default/files/geowebs/OLsurveys/index.htm). The seismic images generated for this study have been deposited in the figshare database under accession code 10.6084/m9.figshare.19919140.
