# Supplementary material for: Evidence for a developing plate boundary in the western Mediterranean
Source: Nat Commun. 2022 Aug 15;13:4786. doi: 10.1038/s41467-022-31895-z (PMC9378692; doi:10.1038/s41467-022-31895-z)
Supplement: Supplementary file 1 — Supplementary Information [file 41467_2022_31895_MOESM1_ESM.pdf]

Evidence for a developing plate boundary in the western Mediterranean

Laura Gómez de la Peña[1*], César R. Ranero[1,2], Eulàlia Gràcia[1], Guillermo Booth-Rea[3,4], José Miguel Azañón[3,4], Umberta Tinivella[5], Abdelkarim Yelles-Chaouche[6]

[1]Barcelona Center for Subsurface Imaging, Instituto de Ciencias del Mar, CSIC, Barcelona, Spain.
[2]ICREA, Barcelona, Spain.
[3]Facultad de Ciencias, Universidad de Granada, Granada, Spain.
[4]Instituto Andaluz de Ciencias de la Tierra, CSIC, Granada, Spain.
[5]Istituto Nazionale di Ocenagrafia e di Geofisica Sperimentale, Trieste, Italy.
[6]Centre de Recherche en Astronomie, Astrophysique et Géophysique, Algiers, Algeria.

*lgomez@icm.csic.de

Supplementary Information

- Supplementary Figures 1 and 2
- Maximum earthquake magnitude estimations

Supplementary Figures

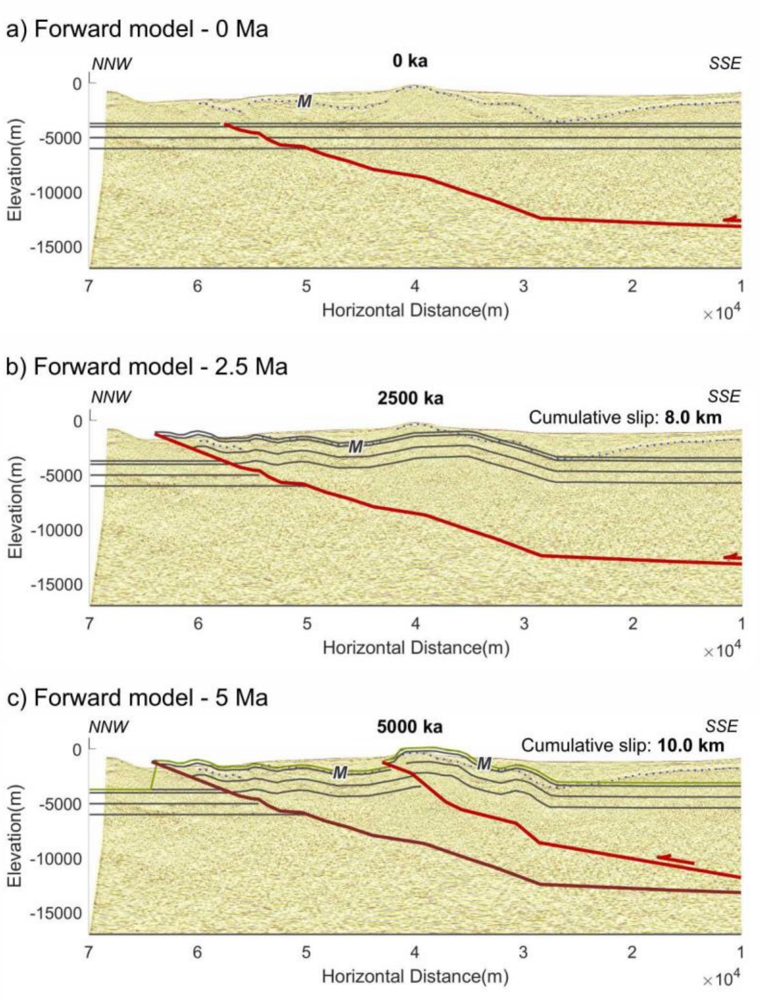

Supplementary Figure 1. Result of the forward modelling for the ARFS. Initial horizontal layers and 2 faults forward model. Years of activity are shown in the upper part of each panel (a-c). When considering horizontal pre-kinematic layers, we need at least one fault splaying from the plate boundary thrust to explain the observed deformation: the plate boundary thrust fits the frontal deformation and the splay under the Alboran Ridge explains the height. Some features cannot be fully modelled, and the northwards dip of the SW layers is not represented (c), perhaps indicating minor back-thrusting.

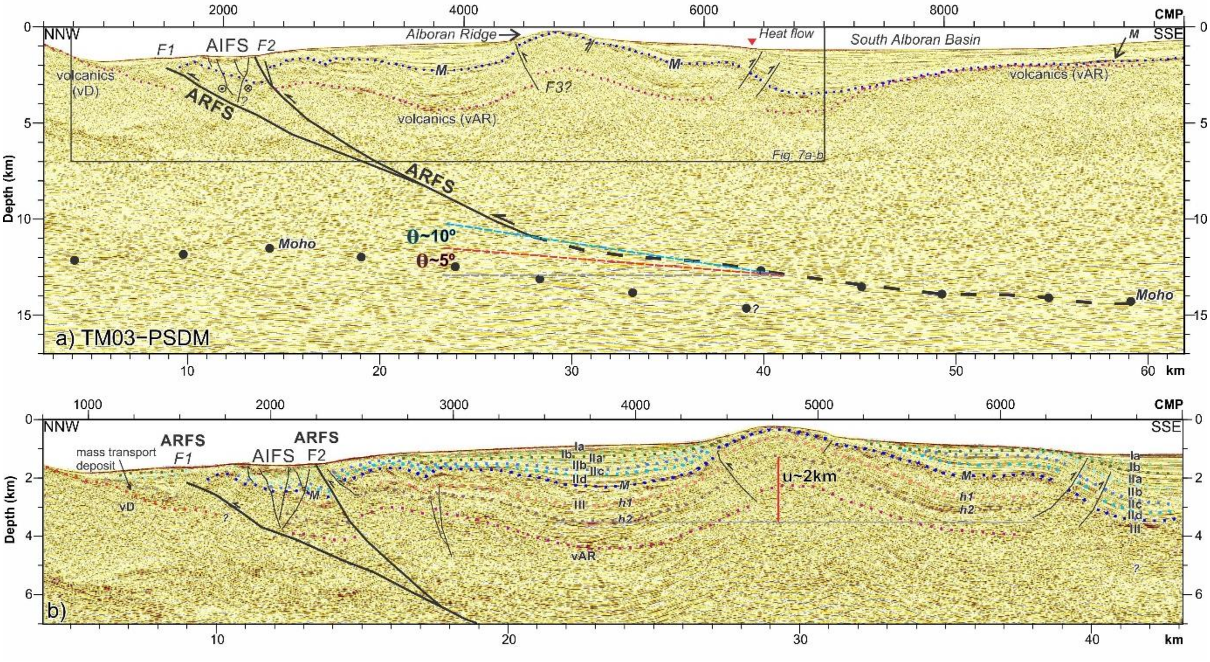

Supplementary Figure 2. The Bend-Fault Folding method applied to the ARFS. a) Pre-stack depth migrated section across the Alboran Ridge (same as Figure 6, see Figures 1 and 2 for location). A detachment angle between 5° and 10º is interpreted. ARFS: Alboran Ridge Fault System, AIFS: Al-Idrissi Fault System, M: Messinian unconformity. **b)** Close up of the shallow structure, in order to measure the maximum uplift (same as Figure 7a). A detachment angle of 5º results in ~23 km of slip and a detachment angle of 10º results in ~11.5 km of slip. d=slip, u=uplift, Ɵ=detachment angle.

Maximum earthquake magnitude estimations

We estimated the maximum magnitude earthquake for each fault using the relations proposed by Wesnouky (2008)[61], based on the length of the fault. For the complex case of the YFS-ARFS rupturing together, we considered both, the strike-slip and thrust relations, although the kinematic of this rupture should be further characterized.

Fault System	Length (km)	Estimated maximum Mw
YFS	160	7.4
ARFS	130	8.0
YFS+ARFS (strike-slip)	300	7.7
YFS+ARFS (reverse)	300	8.8

Wesnousky (2008)[1]: Strike-slip $Mw = 5.56 + 0.87 \log L$;

Reverse $Mw = 4.11 + 1.88 \log L$

Supplementary Table 1: Maximum magnitude earthquake estimations, based on the empirical relationships proposed by Wesnousky (2008)[1] for continental crust.

References:

1. Wesnousky, S. G. Displacement and geometrical characteristics of earthquake surface ruptures: Issues and implications for seismic-hazard analysis and the process of earthquake rupture. *Bull. Seismol. Soc. Am.* **98**, 1609–1632 (2008).
